# Förster resonance energy transfer efficiency of the vinculin tension sensor in cultured primary cortical neuronal growth cones

**DOI:** 10.1117/1.NPh.9.2.025002

**Published:** 2022-05-30

**Authors:** Marina A. Ayad, Timothy Mahon, Mihir Patel, Marina M. Cararo-Lopes, Ilker Hacihaliloglu, Bonnie L. Firestein, Nada N. Boustany

**Affiliations:** a Rutgers University, Department of Biomedical Engineering, Piscataway, New Jersey, United States; b Rutgers University, Department of Cell Biology and Neuroscience, Piscataway, New Jersey, United States

**Keywords:** fluorescence microscopy, Förster resonance energy transfer, growth cones, neurons, vinculin tension sensor

## Abstract

**Significance:**

Interaction of neurons with their extracellular environment and the mechanical forces at focal adhesions and synaptic junctions play important roles in neuronal development.

**Aim:**

To advance studies of mechanotransduction, we demonstrate the use of the vinculin tension sensor (VinTS) in primary cultures of cortical neurons. VinTS consists of TS module (TSMod), a Förster resonance energy transfer (FRET)-based tension sensor, inserted between vinculin’s head and tail. FRET efficiency decreases with increased tension across vinculin.

**Approach:**

Primary cortical neurons cultured on glass coverslips coated with poly-d-lysine and laminin were transfected with plasmids encoding untargeted TSMod, VinTS, or tail-less vinculinTS (VinTL) lacking the actin-binding domain. The neurons were imaged between day *in vitro* (DIV) 5 to 8. We detail the image processing steps for calculation of FRET efficiency and use this system to investigate the expression and FRET efficiency of VinTS in growth cones.

**Results:**

The distribution of fluorescent constructs was similar within growth cones at DIV 5 to 8. The mean FRET efficiency of TSMod (28.5±3.6%) in growth cones was higher than the mean FRET efficiency of VinTS (24.6±2%) and VinTL (25.8±1.8%) ($p<10^{-6}$). While small, the difference between the FRET efficiency of VinTS and VinTL was statistically significant ($p<10^{-3}$), suggesting that vinculin is under low tension in growth cones. Two-hour treatment with the Rho-associated kinase inhibitor Y-27632 did not affect the mean FRET efficiency. Growth cones exhibited dynamic changes in morphology as observed by time-lapse imaging. VinTS FRET efficiency showed greater variance than TSMod FRET efficiency as a function of time, suggesting a greater dependence of VinTS FRET efficiency on growth cone dynamics compared with TSMod.

**Conclusions:**

The results demonstrate the feasibility of using VinTS to probe the function of vinculin in neuronal growth cones and provide a foundation for studies of mechanotransduction in neurons using this tension probe.

## 1 Introduction

Extracellular mechanical cues contribute to cell differentiation, proliferation, and migration. In neurons, these mechanical cues play a role in dictating neuronal morphology and dendritic branching.^1^ In addition, various neural tissue pathologies, such as traumatic brain injury and degenerative diseases, involve alterations in tissue stiffness and expose neurons to environments with altered mechanical properties. Thus, understanding the role of mechanical forces in neuronal development and connectivity may help elucidate mechanotransduction pathways that could be exploited to enhance neuronal repair or regeneration.

Several techniques have been advanced to investigate cellular mechanics. Traction force microscopy has been a technique of choice to measure forces at the cellular level, producing measurements of cellular traction stresses, including their local magnitude and direction.^2^^–^^5^ Molecular fluorescent probes have been developed to sense forces. Some of these probes are expressed on the surface of cells and can sense forces between cells and the substrate to which the cells are attached.^6^^–^^10^ Another class of probes are expressed intracellularly,^11^^–^^14^ such as vinculin tension sensor (VinTS), the probe used in this study,^12^ and have been extensively reviewed.^15^^–^^20^ The purpose of these probes is to measure tension across a specific molecule, such as vinculin, and help elucidate the role of mechanical forces during distinct cellular behaviors. In general, these probes target load-bearing molecules within the cell–substrate or cell–cell adhesion complexes. These sensors have been utilized successfully in epithelial cells where they have shed light on tension-driven regulation of focal adhesions^21^ and adherens junctions.^14^^,^^22^ In neuronal cells, the use of molecular tension probes has been relatively limited. A molecular probe was developed to measure actin tension and axonal growth in response to nerve growth factor and glial scar inhibitors in pheochromocytoma 12 (PC12) cells and to study the role of talin and E-cadherin in this response.^23^ In results reported in a manuscript posted to BioRxiv, a Förster resonance energy transfer (FRET)-based sensor was also developed to measure tension across N-cadherin at synaptic junctions.^24^

In this paper, we demonstrate the use of VinTS^12^ in cortical neurons. VinTS is composed of a FRET tension module, TS module (TSMod), inserted between the vinculin head and tail. TSMod consists of a 40 amino acid flagelliform peptide inserted between monomeric teal fluorescent protein [monomeric teal fluorescent protein 1 (mTFP1)] and mVenus and is sensitive to molecular forces between 1 and 6 pN.^12^ Increased tension across VinTS leads to greater separation between the vinculin head and tail and lower FRET efficiency, reflecting lower quenching of the donor (mTFP1) fluorescence by the acceptor (mVenus).

We chose VinTS as vinculin is a known key regulator of focal adhesions,^12^^,^^25^ and recently, there has been increased focus on investigation of the role and distribution of vinculin in neurons.^26^^–^^29^ The key role of vinculin as a mechanical clutch molecule in adhesion signaling has been well-established in epithelial cells.^30^ Conservation of focal adhesion architecture across the cell and various cell types^31^ suggests that cell adhesion signaling in neurons may involve similar molecules, including vinculin, in response to altered mechanical cues. Indeed, coupling of the cytoskeleton to the substrate affects neuronal growth dynamics.^32^ Vinculin is found in growth cones^33^ and plays a role in filopodial development.^29^ Vinculin co-localizes with focal adhesion kinase (FAK) in neurons.^26^ Furthermore, neurites in vinculin-deficient PC12 are shorter than neurites in control PC12 cells,^28^ and micro-scale chromophore-assisted laser inactivation of vinculin results in bending and buckling of filopodia.^29^ In addition, the force-dependent interaction of vinculin with alpha-catenin^34^ suggests that vinculin may play a role in cadherin-mediated dynamics and dendritic spine formation.^35^ Thus, vinculin is emerging as a significant player in neuronal mechanosensing and adhesion-dependent dynamics.

Here, we measure the FRET efficiency of VinTS in the growth cones of cortical neurons cultured on glass coverslips coated with poly-d-lysine (PDL) and laminin. By comparing the mean FRET efficiency of VinTS to that of TSMod and tail-less vinculinTS (VinTL) that cannot bind actin, our results suggest that between days *in vitro* (DIV) 5 to 8, vinculin is under low tension in the growth cones of neurons. We also observed dynamic changes in VinTS FRET efficiency as a function of time, suggesting that vinculin tension varies within growth cones undergoing morphological changes. Our results provide a basis for further investigations of this tension probe in neurons.

## 2 Methods

### 2.1 Primary Neuronal Culture

Cortices were dissected from embryonic rat brains at gestational day 18 as previously described^36^^,^^37^ and in accordance with protocols approved by the Rutgers Animal Care and Facilities Committee and the National Institutes of Health guide for the care and use of Laboratory animals (Rutgers Institutional AAALAC Accreditation Number: 000534). Tissue was triturated, and neurons were dissociated and cultured. The neuronal culture was maintained at 37°C and 5% ${\mathrm{CO}}_{2}$ in neurobasal medium supplemented with 2% B27 and 1% Glutamax (Invitrogen). The neurons were cultured at a density of $50,000\;\text{cell}/{\mathrm{cm}}^{2}$ on glass coverslips coated with PDL ($0.0025\;\mathrm{mg}/{\mathrm{cm}}^{2}$) and laminin ($0.00025\;\mathrm{mg}/{\mathrm{cm}}^{2}$).

### 2.2 Neuronal Transfection

Transfection with plasmids encoding Vinculin TS (VinTS), TSMod, or VinTL was performed between DIV 4 to 7 for imaging of neurons between DIV 5 to 8 when growth cones can be observed. The transfection was achieved by one hour incubation of the neurons in each well of a 12 well plate in complete Neurobasal medium containing 1.5-μg deoxyribonucleic acid (DNA) plasmid, 1.5-μL PLUS reagent, and 2-μL Lipofectamine LTX (Invitrogen). VinTS, VinTL, and TSMod plasmids were a gift from Martin Schwartz;^12^ VinculinTS (Addgene plasmid #26019;^38^ RRID:Addgene_26019) VinTL (Addgene plasmid #26020;^39^ RRID:Addgene_26020) and TSMod (Addgene plasmid #26021^40^ RRID:Addgene_26021).

### 2.3 Treatment with Y-27632 (Y Compound)

For treatment with the Rho-associated kinase (ROCK) inhibitor Y-27632, the aqueous stock solution (5 mM) of Y-27632 (Cat. #688001, Sigma-Aldrich, St. Louis, Missouri, United States) was dissolved in full Neurobasal medium to a final concentration of 10-μM. Neurons expressing VinTS or VinTL were incubated at 37°C and 5% ${\mathrm{CO}}_{2}$ for 2 h in full neurobasal medium containing either 10-μM Y-27632 or sterile water (vehicle control). Following incubation, the medium was switched to HEPES-buffered balanced salt solution (HBBSS^41^) for live cell imaging.

### 2.4 Image Acquisition

Imaging was performed at 24 to 48 h after transfection. Each glass coverslip was mounted onto a custom-made metal slide chamber filled with HEPES-buffered balanced salt solution (HBBSS^41^) and was imaged on an automated wide-field epi-fluorescence microscope (Zeiss Axiovert 200 M) using a 63× oil immersion objective with a 1.4 numerical aperture (Zeiss plan Apochromat 63× 1.4 oil, 440762-9904). Initially, images of growth cones from neurons expressing VinTS and TSMod were collected on an electron multiplying charge-coupled device (EM-CCD) camera (Cascade 512B, Photometrics, Roper Scientific) operated by the IPLab 4.0.8 software (Becton Dickinson, BD Biosciences). Throughout the project, the system was upgraded, and additional images of growth cones from neurons expressing VinTS, VinTL, or TSMod were collected on a scientific complementary metal oxide semiconductor (sCMOS) camera (PCO Edge 4.2bi, PCO, GmbH). The sCMOS was operated by VisiView software (Visitron Systems GmbH). The microscope was equipped with a mercury arc source and excitation and emission filter wheels (Ludl) mounted with filters for the mTFP1/mVenus FRET pair: mTFP1 excitation filter (Chroma, ET450/30×), Venus excitation filter (Chroma, ET514/10×), mTFP1 emission filter (Chroma, ET485/25 m), Venus emission filter (Semrock, FF01-571/72). The excitation and emission filters were used with a single two-band dichroic mirror (Chroma, T450/514rpc) mounted in the filter reflector turret of the microscope. To minimize photobleaching, two neutral density filters with a total optical density = 1.5 were placed in front of the excitation source.

We measured FRET efficiency using the conventional three cube method, which consists of acquiring images in three channels: (1) the donor channel ($I_{\mathrm{DD}}$) with mTFP1 excitation and emission bandwidths centered at 450 and 485 nm, respectively; (2) the acceptor channel ($I_{\mathrm{AA}}$) with mVenus excitation and emission bandwidths centered at 514 and 571 nm, respectively; (3) the raw FRET channel ($I_{\mathrm{DA}}$) with mTFP1 excitation centered at 450 nm and mVenus emission centered at 571 nm. All three images $I_{\mathrm{DD}}$, $I_{\mathrm{AA}}$, and $I_{\mathrm{DA}}$ were acquired with the same exposure time.

### 2.5 Image Processing

**Fig. 1:**
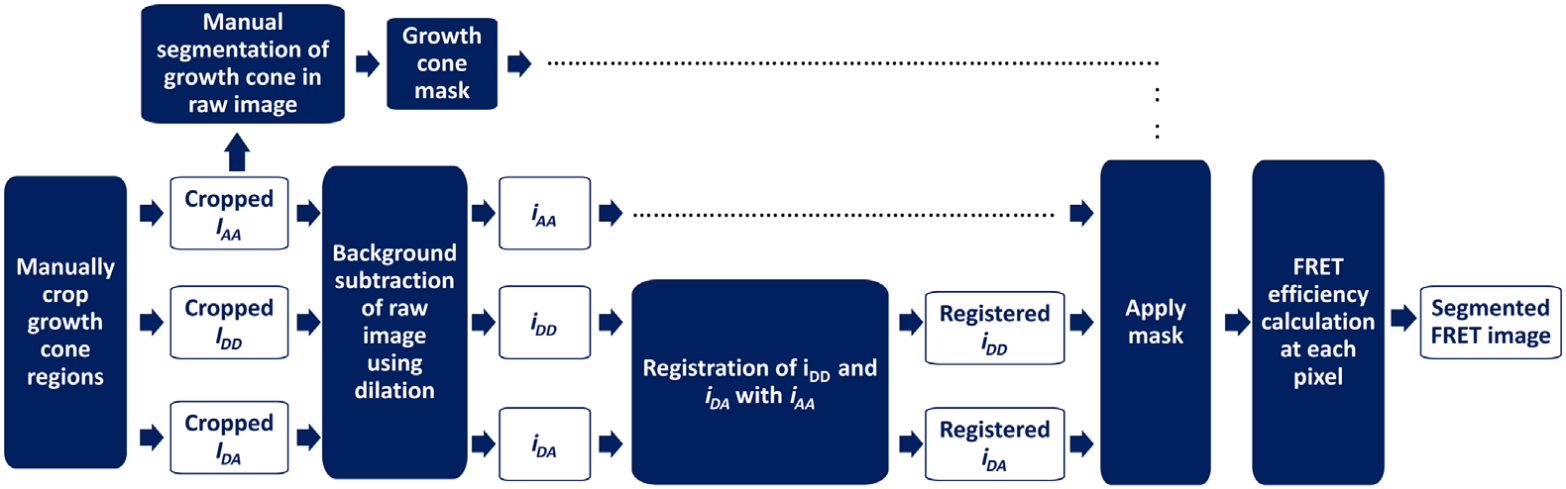
Image processing flowchart to calculate the FRET efficiency in the growth cones images. $I_{\mathrm{AA}}$, $I_{\mathrm{DD}}$, and $I_{\mathrm{DA}}$ denote the raw images in the three channels; $i_{\mathrm{AA}}$, $i_{\mathrm{DD}}$, and $i_{\mathrm{DA}}$ denote the background-subtracted cropped images. Manual segmentation was performed on the cropped raw $I_{\mathrm{AA}}$ image. The resulting mask was used to limit the FRET efficiency calculation to the pixels within the growth cone region.

Several image processing steps were undertaken before calculating FRET efficiency, as shown in Fig. 1.

#### 2.5.1 Background subtraction

The growth cones images were cropped from the raw images, and the cropped images were background subtracted. On the EM-CCD with magnification of 0.25-μm per pixel, images 96  pixels×96  pixels in size were cropped from the 512  pixels×512  pixels raw images. On the sCMOS with magnification of 0.1  μm per pixel, images 240  pixels×240  pixels in size were cropped from the 2048  pixels×2048  pixels raw images. The field of view of the cropped images was $24\times 24\;\mu m^{2}$. In each of the three acquisition channels, the background was estimated in the cropped image by gray-scale morphological opening (“imopen” function in MATLAB, The Mathworks, Inc., Natick, Massachusetts, United States). Morphological opening consists of image erosion followed by dilation with a “rolling” structuring element of specified shape and size over the image.^42^ When the structuring element size is larger than the objects in the foreground of the image (the growth cones in our case), morphological opening assigns the minimum pixel value (value in the background regions surrounding the growth cone) to the pixels within the structuring element as it is translated over the image. The resulting opened image, which gives a local estimate of the background away from the growth cone, is then subtracted from the original cropped image. The structuring element was a disk of radius 50 pixels on the EM-CCD images; a disk element of radius 125 pixels on the sCMOS. The deployed structuring elements were kept fixed and were not changed throughout the analyses. The radial length of the growth cones typically varied in the range of 1.25 to 7.5  μm.

#### 2.5.2 Image registration and segmentation

Image registration was achieved using multimodal rigid registration (EM-CCD) or translation (sCMOS), based on optimization of mutual information.^43^ This was done in MATLAB (The Mathworks, Inc.) using the “imregister” function with the cropped $I_{\mathrm{AA}}$ as the reference image and cropped $I_{\mathrm{DD}}$ or $I_{\mathrm{DA}}$ as the moving image. We found that the $I_{\mathrm{AA}}$ and $I_{\mathrm{DA}}$ images were initially registered for the data collected on the EM-CCD. However, both $I_{\mathrm{DA}}$ and $I_{\mathrm{DD}}$ required registration on the sCMOS. To check the similarity of the registered images, the registered images were binarized and compared using the Sørensen–Dice similarity coefficient (“Dice” function in MATLAB, The Mathworks, Inc.).^44^^,^^45^ For two binary image inputs ${i^{\prime }}_{\mathrm{DX}}$ and ${i^{\prime }}_{\mathrm{AA}}$, the Dice similarity coefficient is given by $2*|i_{\mathrm{DX}}^{\prime }\;\cap i_{\mathrm{AA}}^{\prime }|/(|i_{\mathrm{DX}}^{\prime }|+|i_{\mathrm{AA}}^{\prime }|)$ and can take values between 1 (similar) and 0 (not similar).^46^ “Dice” calculates the number of overlapping pixels between the two input images divided by the number non-zero pixels in the input images. It is a measure of spatial overlap between two images that is commonly used for segmentation and registration verification.^46^ The Dice coefficient was >0.99 for all registered images considered for further analysis. The images were manually segmented to limit the analysis of FRET efficiency to the growth cones region without including the background pixels in the calculations. The segmentation was performed on the copped $I_{\mathrm{AA}}$ image and the resulting masks were subsequently applied to all three registered channels (Fig. 1).

#### 2.5.3 FRET efficiency calculation

A FRET efficiency image was generated by calculating FRET efficiency at each pixel of the segmented images as follows:

$$E=\frac{F_{c}/i_{\mathrm{DD}}}{G+\;F_{c}/i_{\mathrm{DD}}},$$ (1)

where $i_{\mathrm{DD}}$ is the donor channel image after background subtraction and registration. $F_{c}$ is the corrected FRET image obtained by subtracting the donor and acceptor bleed-through from the $I_{\mathrm{DA}}$ image as explained by Menaesse et al.^47^ with $F_{c}=i_{\mathrm{DA}}-a\cdot i_{\mathrm{AA}}-d\cdot i_{\mathrm{DD}}$. $i_{\mathrm{AA}}$ and $i_{\mathrm{DA}}$ are the background-subtracted and registered acceptor channel and FRET channel image, respectively. The G-factor is a calibration constant for a specific imaging setup and fluorophore pair. The G-factor was obtained in separate calibration experiments using measurements of $F_{c}$ and $i_{\mathrm{DD}}$ and the known FRET efficiency of TSMod, as explained in detail by Menaesse et al.^47^ For this calibration, the TSMod FRET efficiency was taken as 0.286, based on data from Gates et al.^48^ We measured bleed-through values of a=0.48 and d=0.84 for the EM-CCD; a=0.47 and d=0.81 for the sCMOS. G was 2.08 for the EM-CCD; 3.15 for the sCMOS.

## 3 Results

### 3.1 Generation of Registered and Segmented Growth Cones images

**Fig. 2:**
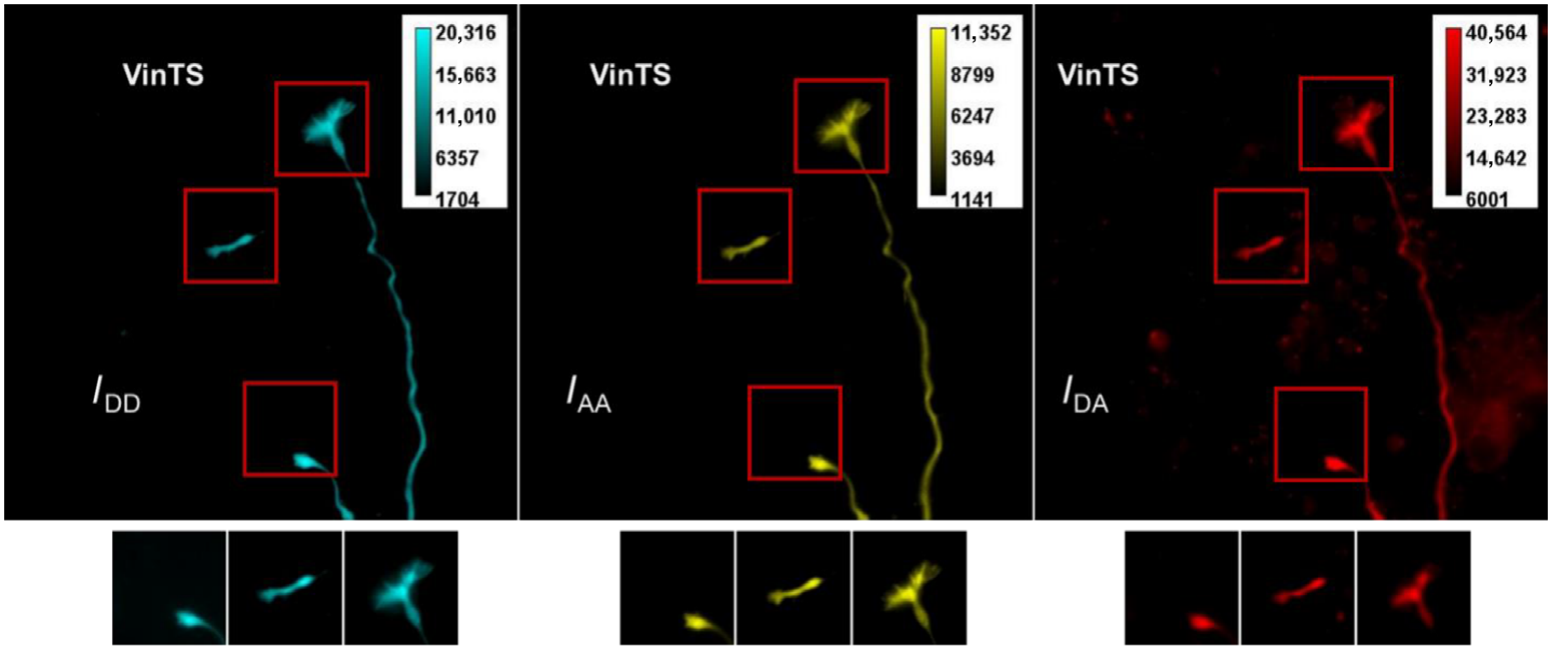
Raw images of growth cones expressing VinTS. The three images are the output of three imaging channels: donor (${\mathrm{mTFP}}_{1}$) excitation and donor emission, $I_{\mathrm{DD}}$ (cyan); acceptor (mVenus) excitation and acceptor emission, $I_{\mathrm{AA}}$ (yellow); FRET channel, donor excitation, and acceptor emission, $I_{\mathrm{DA}}$ (red). The original field of view is 128×128  μm (EM-CCD). Growth cones regions (red boxes) were selected for processing to calculate FRET efficiency. Under each original image are the corresponding selected growth cone areas. The field of view in the cropped regions with the growth cones is $24\times 24\;\mu m^{2}$.

**Fig. 3:**
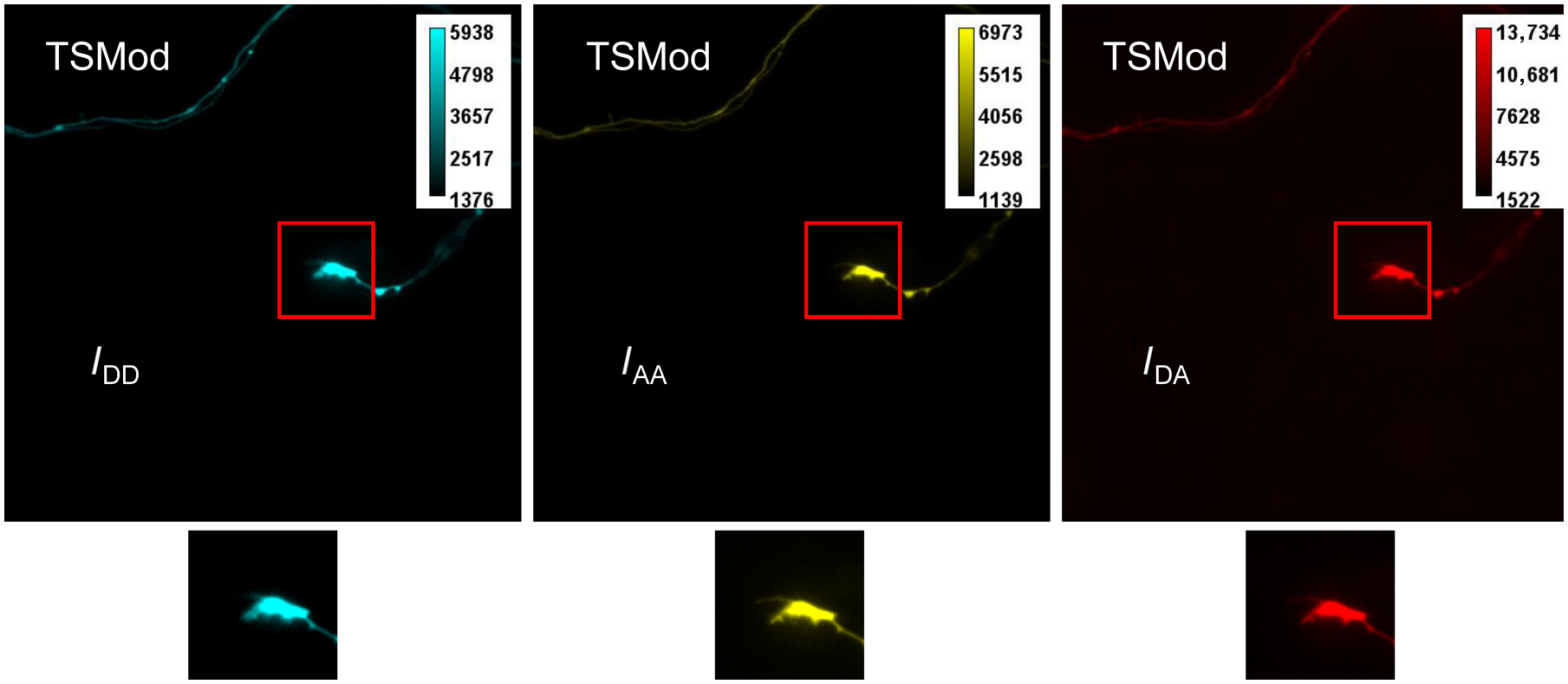
Raw images of growth cones expressing TSMod. The three images are the output of three imaging channels: donor (${\mathrm{mTFP}}_{1}$) excitation and donor emission, $I_{\mathrm{DD}}$ (Cyan); acceptor (mVenus) excitation and acceptor emission, $I_{\mathrm{AA}}$ (yellow); FRET channel, donor excitation and acceptor emission, $I_{\mathrm{DA}}$ (red). The original field of view is 128×128  μm (EM-CCD). A growth cone region (red box) was selected for processing to calculate FRET efficiency. Under each original image is the corresponding selected growth cone area. The field of view in the cropped region with the growth cone is $24\times 24\;\mu m^{2}$.

Representative raw images in the three channels, $I_{\mathrm{DD}}$ (donor channel, cyan), $I_{\mathrm{AA}}$ (acceptor channel, yellow), and $I_{\mathrm{DA}}$ (FRET channel, red), are shown for a neuron expressing VinTS (Fig. 2) and a neuron expressing TSMod (Fig. 3). For subsequent FRET efficiency calculations, regions with growth cones were identified in the raw images and cropped as shown in Fig. 2 for three growth cones expressing VinTS and in Fig. 3 for one growth cone expressing TSMod.

**Fig. 4:**
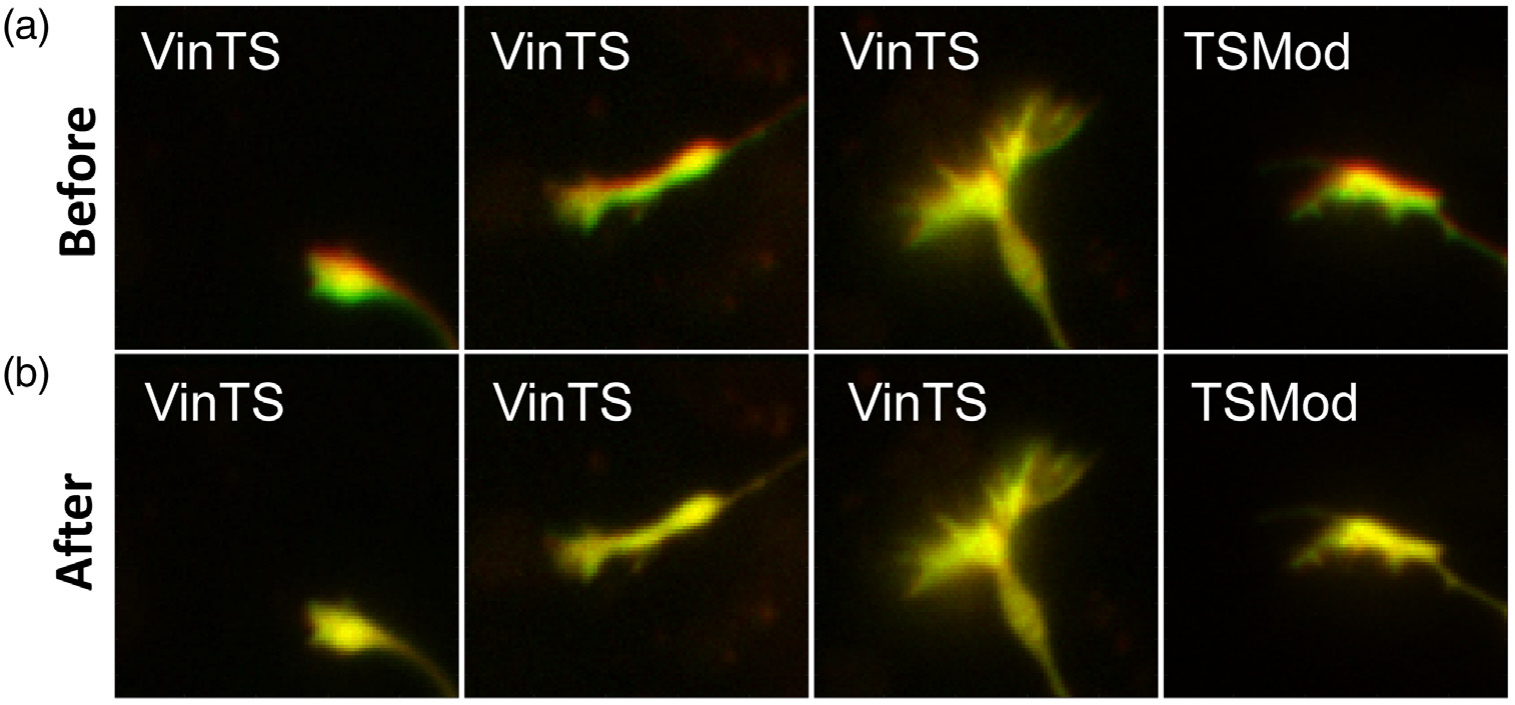
Registration of the $I_{\mathrm{DD}}$ image (red) with the $I_{\mathrm{AA}}$ image (green). Image overlays show the overlap (yellow) between the two images (a) before and (b) after registration for the copped VinTS and TSMod growth cones images selected in Figs. 2 and 3.

**Fig. 5:**
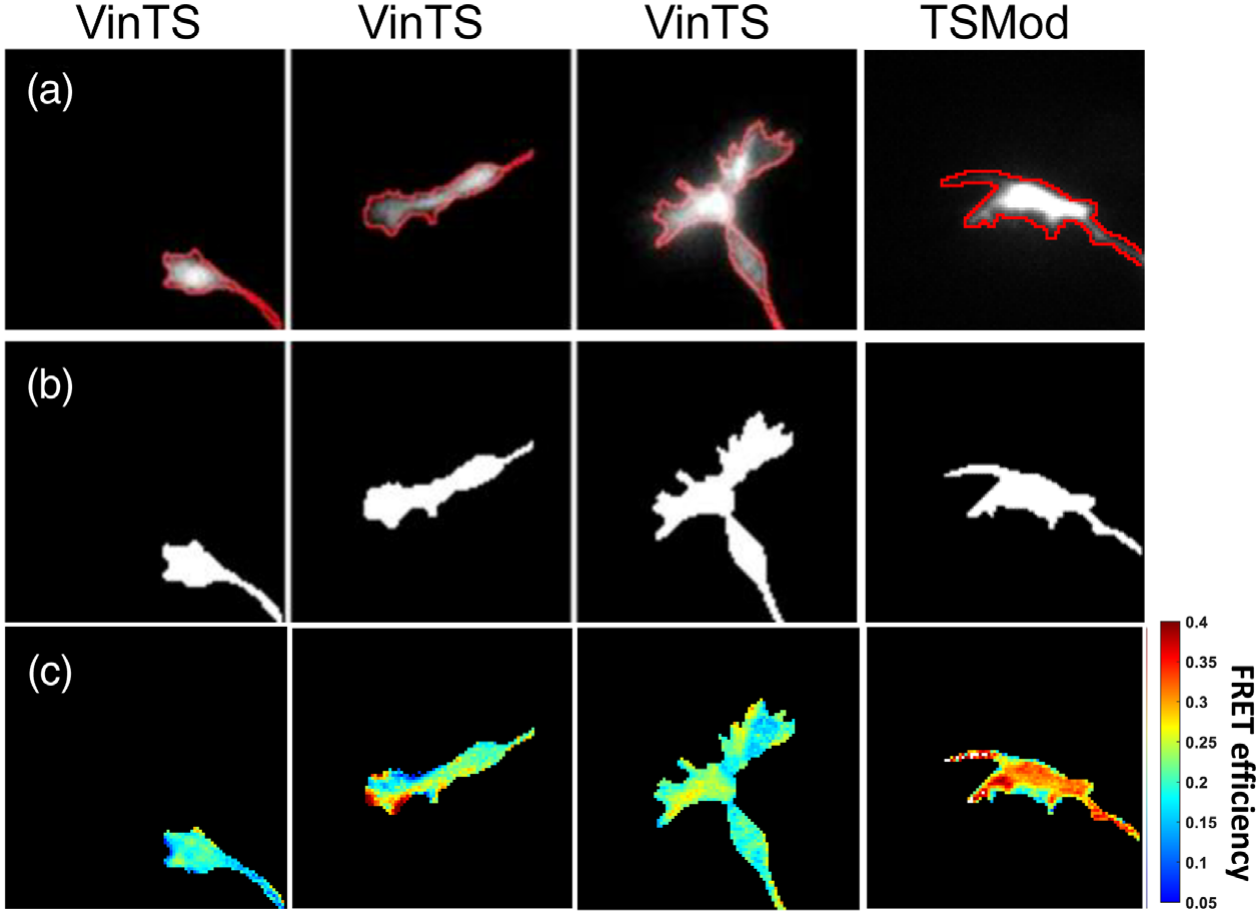
(a) Manually segmented contours of growth cones in the cropped $I_{\mathrm{AA}}$ channel. (b) Binary masks corresponding the segmented regions (white) where the FRET efficiency is calculated. The black regions are excluded from the calculation. (c) Corresponding segmented FRET efficiency images.

Examples of image registration between the $I_{\mathrm{DD}}$ and $I_{\mathrm{AA}}$ channels are shown in Fig. 4. The cropped $I_{\mathrm{AA}}$ image was also manually segmented [Figs. 5(a) and 5(b)]. The segmentation yielded a binary mask, which was applied to the background-subtracted and registered $i_{\mathrm{AA}}$, $i_{\mathrm{DD}}$, and $i_{\mathrm{DA}}$ cropped images as explained in Fig. 1. Calculation of FRET efficiency was therefore limited to the pixels within the segmented mask [Fig. 5(c)].

### 3.2 Analysis of FRET Efficiency at the Growth Cones

**Fig. 6:**
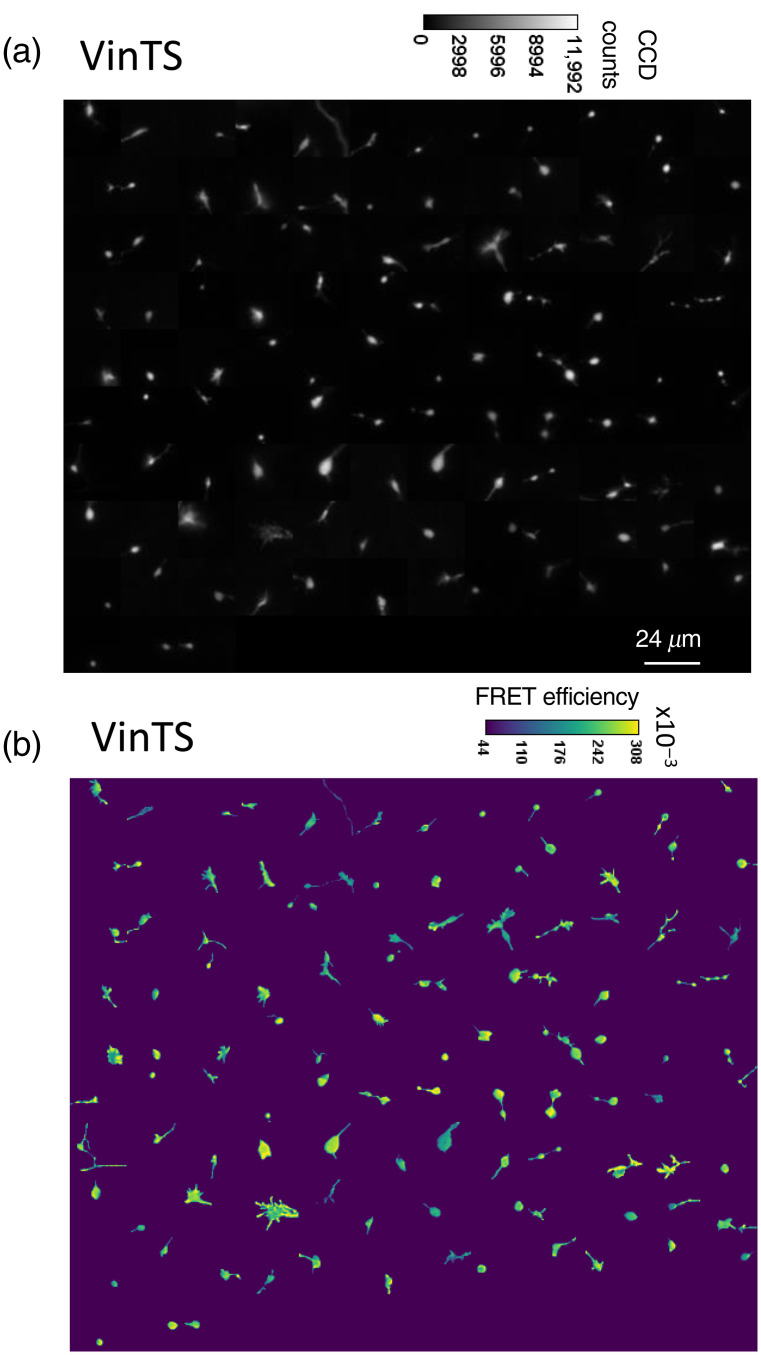
(a) Montage of background subtracted acceptor intensity images and (b) corresponding FRET efficiency images of growth cones expressing VinTS.

**Fig. 7:**
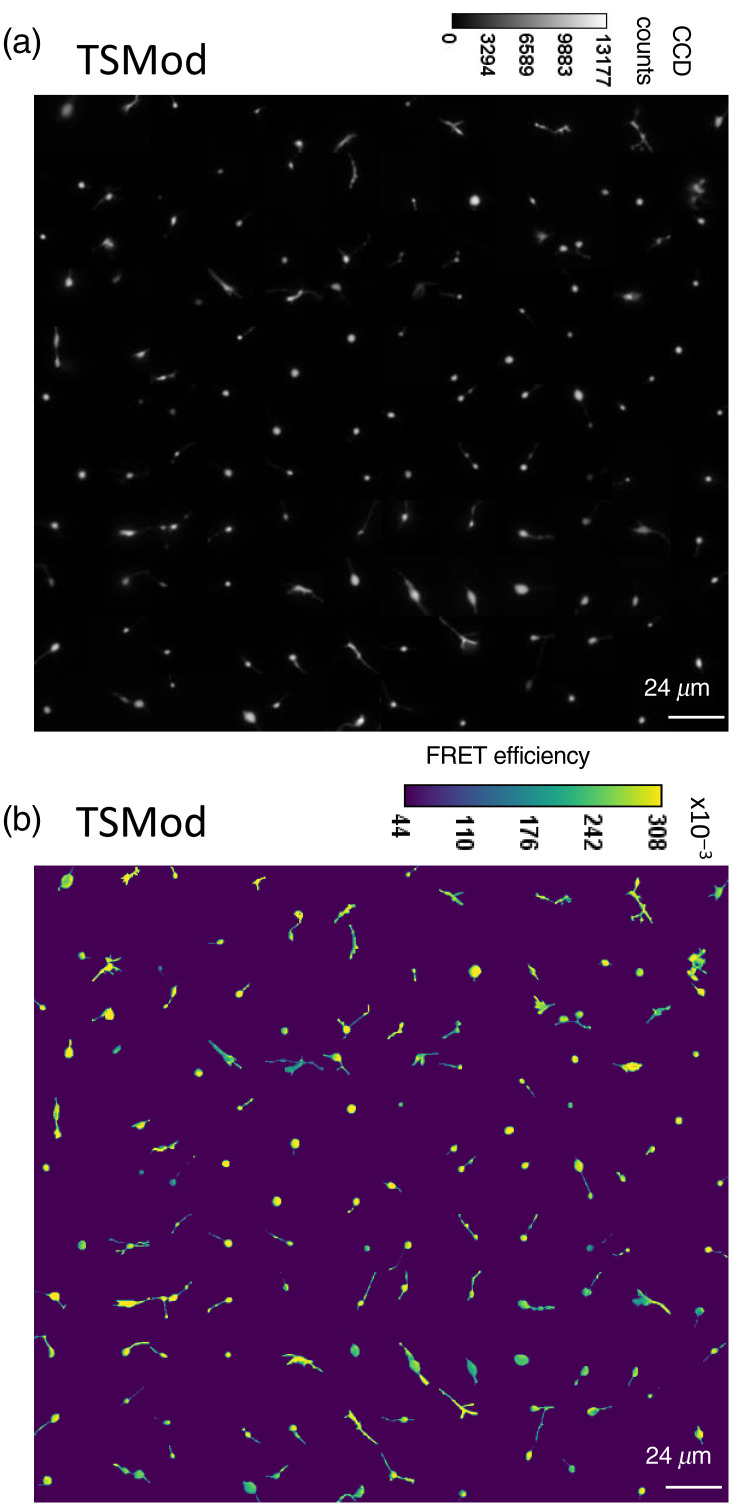
(a) Montage of background subtracted acceptor intensity images and (b) corresponding FRET efficiency images of growth cones expressing TSMod.

**Fig. 8:**
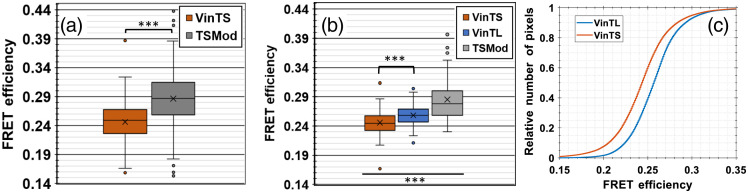
(a) Box and whisker plot of FRET efficiency measurements in growth cones from neurons expressing VinTS and TSMod. *** denotes significance of p<0.001 by Welch’s t-test. The cross (×) denotes the mean. The line denotes the median of the dataset, and the top and bottom of the box denote the 75th and 25th percentile, respectively. The black lines extending from the box (whiskers) corresponds to the maximum and minimum values, which are not considered outliers. Individual points above and below the whiskers are considered outliers with values larger than ±1.5× [the difference between the 75th and 25th percentile]. (b) The experiment in (a) was repeated to include VinTL as an additional control. *** denotes significance of p<0.001 by ANOVA followed by Tukey’s multiple comparisons test. (c) Cumulative distribution functions for pixels from growth cones from neurons expressing VinTS and VinTL [data from (b) only].

Although VinTS was recruited into growth cones, growth cones from neurons expressing VinTS showed no clearly discernable difference in the spatial distribution of VinTS expression compared with TSMod expression [Figs. 6(a) and 7(a)]. By generating the corresponding FRET efficiency images [Figs. 6(b) and 7(b)], we initially investigated whether growth cones that express VinTS exhibit a different FRET efficiency compared with growth cones that express the untargeted and unloaded TSMod. The mean (±standard deviation) FRET efficiency of the growth cones expressing VinTS (n=111 images, N=8 experimental repeats) was 24.6±3.8%, significantly lower ($p=2.69\;10^{-12}$ by Welch’s t-test) than the mean FRET efficiency of growth cones expressing TSMod (n=131 images, N=9 experimental repeats), which was 28.67±4.8% [Fig. 8(a)].

**Fig. 9:**
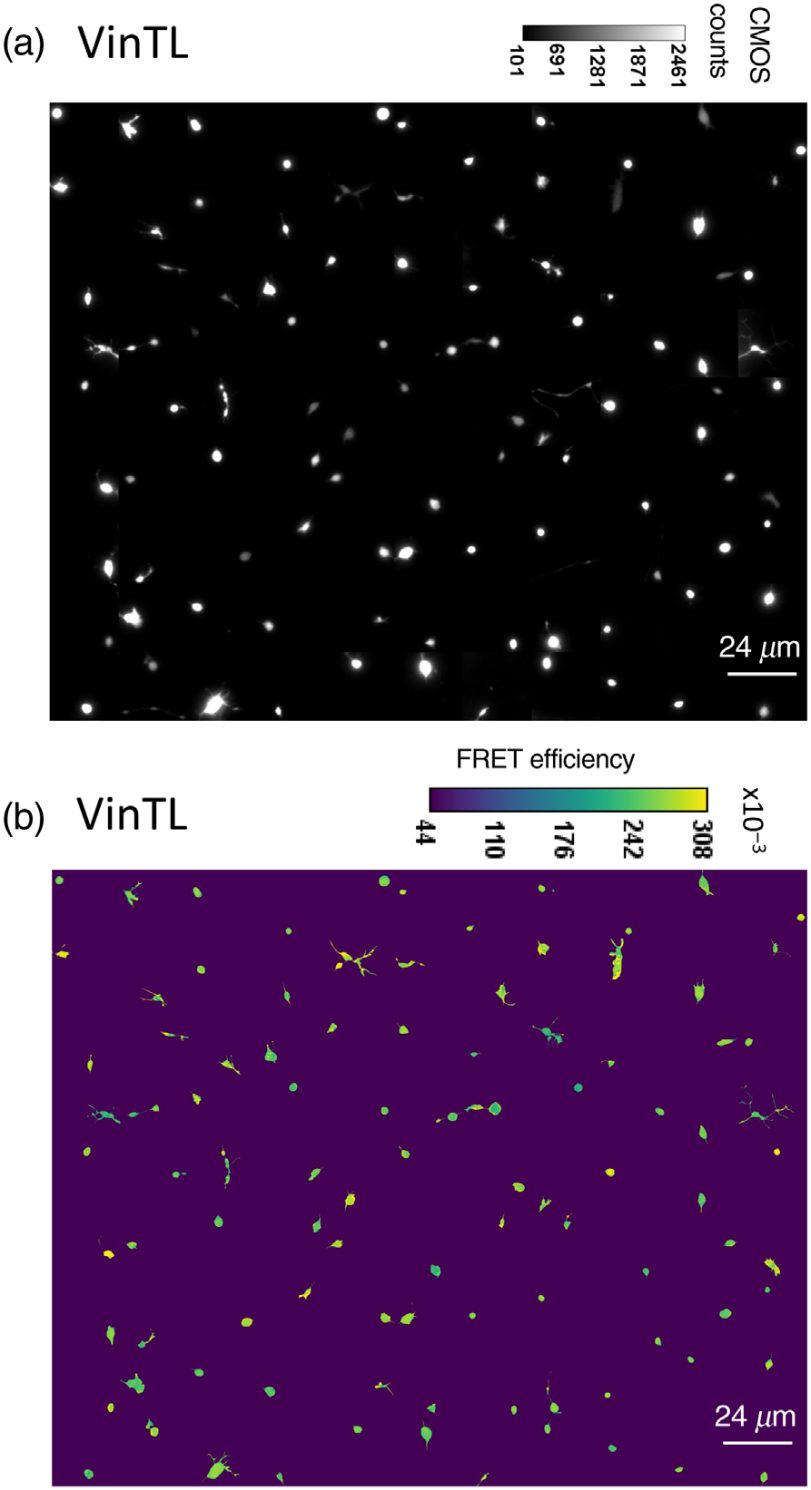
(a) Montage of background subtracted acceptor intensity images and (b) corresponding FRET efficiency images of growth cones expressing VinTL.

To establish whether the measured lower FRET efficiency of VinTS is due to tension in the growth cones, we repeated our experiments with TSMod, VinTS, and VinTL. VinTL lacks the actin-binding domain of vinculin and acts as a negative control in which the tension sensor is targeted to vinculin but should not sense tension.^12^ Representative images of growth cones expressing VinTL are shown in Fig. 9. The mean (± standard deviation) FRET efficiency of both VinTL (25.8±1.8%, n=99 images, N=5 experimental repeats) and VinTS (24.6±2%, n=111 images, N=6 experimental repeats) in the growth cones was lower than the mean FRET efficiency of TSMod (28.5±3.6%, n=68 images, N=3 experimental repeats), suggesting that the FRET efficiency of the tension module is decreased when it is expressed in vinculin ($p<10^{-6}$ determined by one-way analysis of variance (ANOVA) followed by Tukey’s multiple comparisons test). The mean FRET efficiency of VinTS was slightly lower than the mean FRET efficiency of VinTL ($p<10^{-3}$), suggesting that VinTS is under a low level of tension in the growth cones [Fig. 8(b) and violin plot (Fig. S1 in the Supplemental Materials)]. The FRET efficiency of VinTS and TSMod observed in the second experiment [Fig. 8(b)] corroborates the values found in the first set of data [Fig. 8(a)]. A cumulative distribution function of pixels obtained from growth cones expressing VinTS is shifted to lower FRET compared to the distribution of pixels from growth cones expressing VinTL [Fig. 8(c)]. To highlight the spatial contribution of high and low FRET pixels, we show segregated regions of pixels above and below 0.25 FRET for growth cones expressing VinTS and VinTL (Fig. S2 in the Supplemental Materials). The ratio of pixels with FRET efficiency ≥0.25 to pixels with FRET efficiency <0.25 was 0.66 for the growth cones from neurons expressing VinTS compared with 1.55 for those expressing VinTL.

To assess whether the lower FRET efficiency of VinTS could be altered by inhibiting actin-myosin contractility, we treated the neurons with the ROCK inhibitor Y-27632. Y-27632 inhibits myosin II by deactivating the Rho signaling pathway and has been used by others to test the effects of actomyosin contraction on the measured FRET efficiency of VinTS.^12^ Unexpectedly, we found that treatment with 10-μM Y-27632 for 2 h did not affect the FRET efficiency of VinTS expressed in growth cones. Both the average FRET efficiency in the growth cones and the pixel distributions did not change significantly after treatment (Fig. S3 in the Supplemental Materials). A two-way ANOVA followed by Tukey’s multiple comparisons test showed a significant difference between FRET efficiency of the VinTL and VinTS groups ($p=7.84\;10^{-4}$), but no significant difference was observed between the treated and control groups for each construct.

### 3.3 Timelapse Imaging

**Fig. 10:**
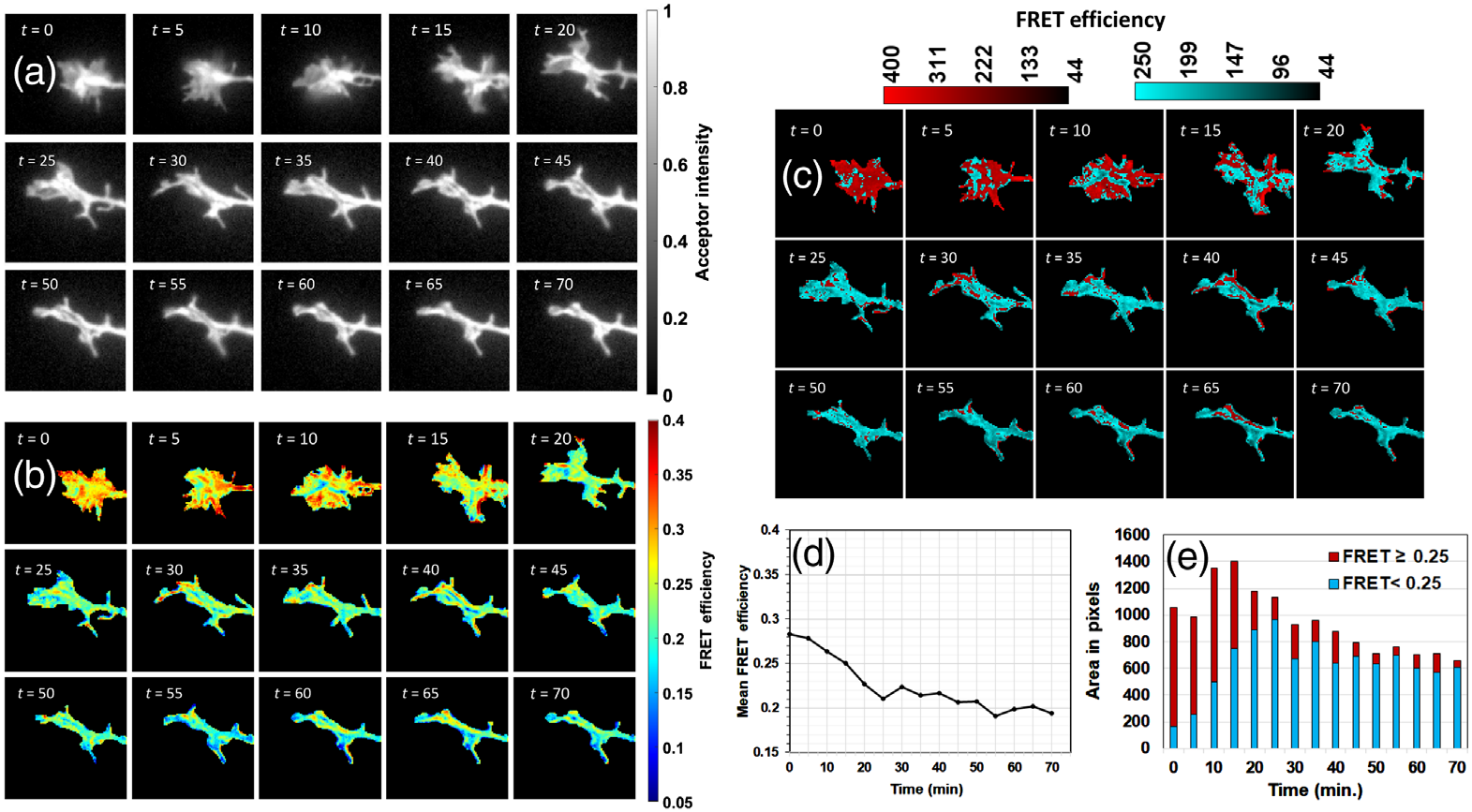
(a) Time-lapse images of background-subtracted and normalized $i_{\mathrm{AA}}$ and (b) FRET efficiency of one growth cone from a neuron expressing VinTS. (c) Pixels with FRET efficiency ≥0.25 (red) and those with FRET efficiency <0.25 (blue) are segregated to highlight the spatial differences in FRET efficiency. (d) Mean FRET efficiency of the growth cone as a function of time. (e) Area fraction of pixels with high (≥0.25) and low (<0.25) FRET efficiency. The field of view is $24\times 24\;\mu m^{2}$ in each image.

**Fig. 11:**
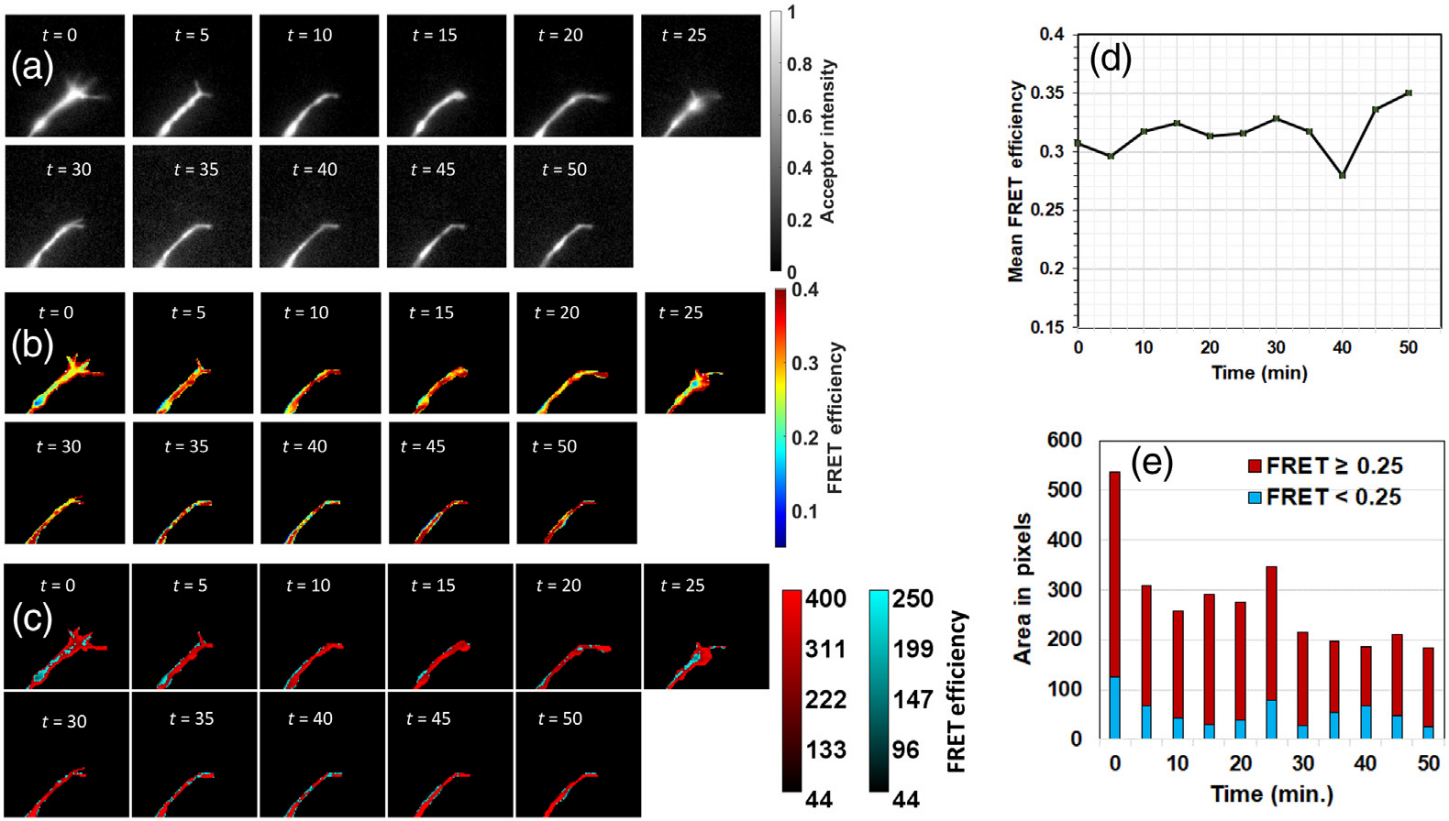
(a) Time-lapse images of background-subtracted and normalized $i_{\mathrm{AA}}$ and (b) FRET efficiency of one growth cone from a neuron expressing TSMod. (c) Pixels with FRET efficiency ≥0.25 (red) and those with FRET efficiency <0.25 (blue) are segregated to highlight the spatial differences in FRET efficiency. (d) Mean FRET efficiency of the growth cone as a function of time. (e) Area fraction of pixels with high (≥0.25) and low (<0.25) FRET efficiency. The field of view is $24\times 24\;\mu m^{2}$ in each image.

The morphology of the growth cones is expected to vary dynamically over time, and the images analyzed thus far likely represent snapshots of this time-dependent behavior. To probe dynamic changes in FRET efficiency, five growth cones expressing either VinTS or TSMod were imaged every 5 min over periods varying between 25 and 70 min. The images were refocused before each capture. In a representative growth cone expressing VinTS (Fig. 10), we observe a rapid change in morphology where filopodia start forming and the FRET efficiency in the growth cone changes as a function of time. To illustrate this change, we constructed color-coded images in which FRET values higher than 0.25 are pseudocolored in red and values under 0.25 are pseudocolored in blue [Fig. 10(c)]. At the beginning of the time lapse sequence, there are both high and low values of FRET efficiency with both red and blue channels clearly visible and intermingled, while only 8% of pixels have low FRET after 30 min. By comparison, in a representative growth cone expressing TSMod (Fig. 11), >60% of pixels have FRET efficiency higher than 0.25 throughout the time series. The mean FRET efficiency of the VinTS sample is 0.28 at time = 0 and drops gradually to ∼0.21 at 50 min., while the mean FRET efficiency of the TSMod sample remains above 0.27 over the 50-min time period.

**Fig. 12:**
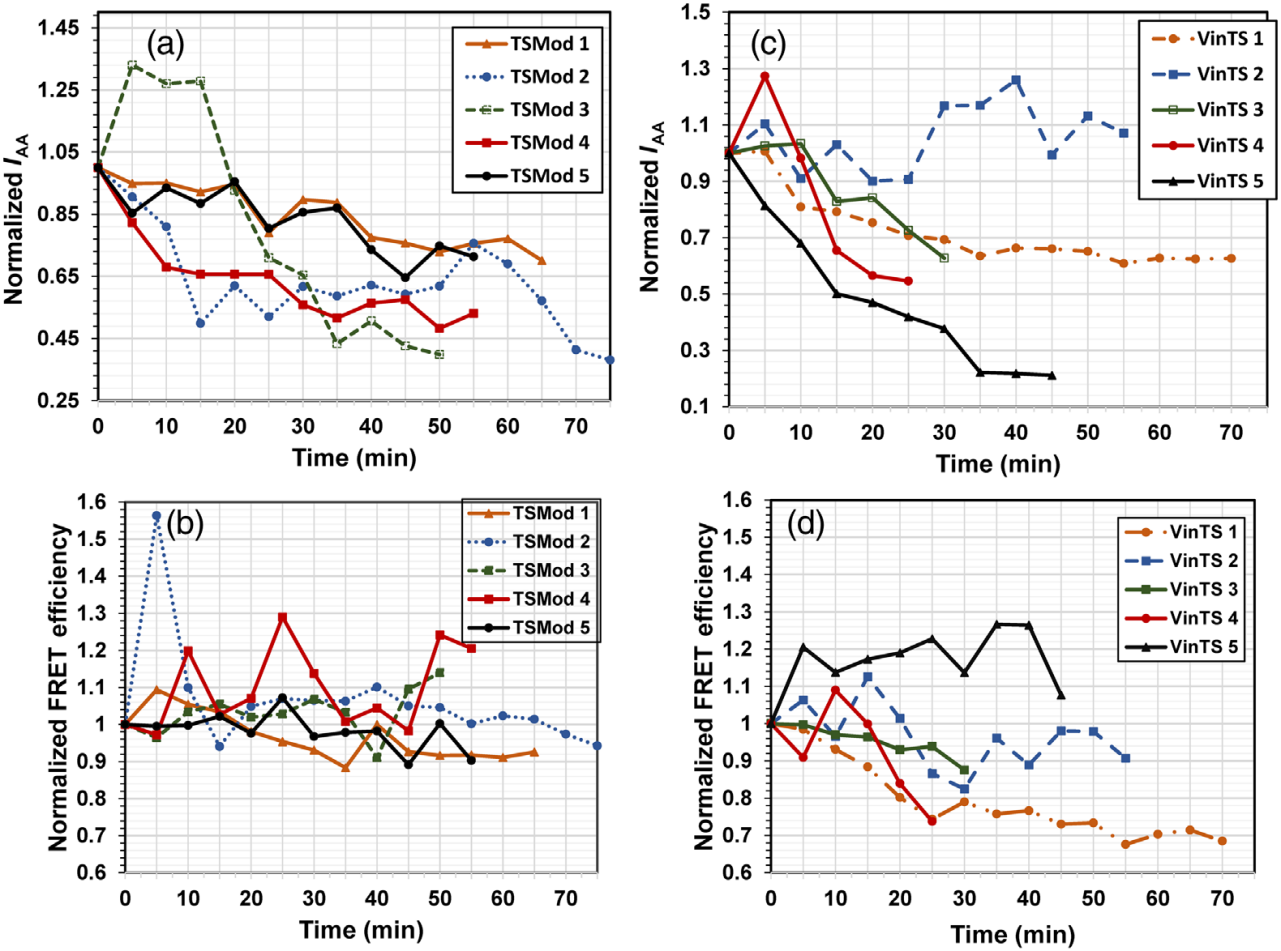
Mean acceptor signal and FRET efficiency normalized to the initial value and plotted over time for five individual growth cones expressing (a) and (b) TSMod or (c) and (d) VinTS. VinTS-1 and TSMod-3 correspond to the samples shown in Figs. 10 and 11, respectively.

For each of the samples tested, the mean acceptor intensity and FRET efficiency were plotted over time after normalization to the initial value (Fig. 12). The variance in FRET efficiency for the samples expressing TSMod was 10% [standard deviation of all the values measured as a function of time for the data series in Fig. 12(b)], compared with 16% for the samples expressing VinTS [data series in Fig. 12(d)], supporting larger variations in VinTS FRET efficiency as a function of growth cone dynamics compared with TSMod. A drop in the value of $I_{\mathrm{AA}}$ at later time points could be due to photobleaching. However, this decrease in $I_{\mathrm{AA}}$ signal was not accompanied by a corresponding drop in TSMod FRET efficiency, indicating that FRET efficiency was not affected by photobleaching at these time points.

## 4 Discussion

We investigated the expression and FRET efficiency of the VinTS in the growth cones of primary rat cortical neurons cultured on glass coverslips coated with PDL and laminin. The neurons were transfected with plasmids encoding either the untargeted tension module TSMod, VinTS, which consists of TSMod inserted between the head and tail of vinculin, or VinTL, consisting of a VinTL lacking the actin-binding domain of vinculin. The probes were expressed in neuronal growth cones at DIV 5 to 8, but they did not clearly localize to punctate regions. The mean FRET efficiency of TSMod (28.5±3.6%) in the growth cones was higher than both the mean FRET efficiency of VinTS (24.6±2%) and VinTL (25.8±1.8%) ($p<10^{-6}$). The FRET efficiency of VinTS was lower than that of VinTL ($p<10^{-3}$). This difference, while statistically significant, was small and suggests that vinculin is under low tension in neurons at DIV 5 to 8. By comparison, a lower VinTS FRET efficiency of 20.5% (and therefore higher tension) was reported at the focal adhesions of live mouse embryonic fibroblasts.^48^ FRET efficiency was not correlated with acceptor signal in the growth cones studied (Fig. S4 in the Supplemental Materials). Thus, the observed decrease in VinTS FRET efficiency is unlikely to be due to a systematic error in our data caused by an artifact related to the level of measured signal.

To further assess the potential source of the decreased FRET efficiency of VinTS compared with VinTL, we treated the cells with the ROCK inhibitor Y-27632. We expected short-term inhibition of myosin II via the Rho signaling pathway to decrease contractility, and therefore, further decrease the tension across vinculin and increase FRET efficiency to the level of VinTL. However, a 2 h treatment with 10-μM Y-27632 did not significantly increase or change the mean FRET efficiency of VinTS in growth cones (Fig. S3 in the Supplemental Materials). In neurons, the roles of actomyosin contractility in developing growth cones and in mediating changes to tension have not been fully elucidated. A balance between neurite outgrowth and contractility appears to exist, and myosin II light chain has been studied as a neurite outgrowth regulator. In hippocampal neurons, inhibition of myosin II by blebbistatin, ML-7, or Y-27632 for 24 to 48 h increased neurite outgrowth as measured by an increase in process length.^49^ Similarly, in PC12 cells, neurite outgrowth increased after 2 or 3 h of treatment with Y-27632.^50^ In the peripheral nervous system, activation of ROCK results in contractility and growth cone retraction.^51^ Although tension has not been directly connected to ROCK inhibition, neurite outgrowth and vinculin distribution were studied in rat hippocampal neurons. Treatment with lysophosphatidic acid, which activates RhoA, followed by Y-27632 treatment enhanced vinculin recruitment to the cell body membrane and promoted microtubule assembly in the growth cones.^52^ Furthermore, other factors, such as cytosolic PSD-95 interactor (cypin), can act downstream of Rho kinase to regulate dendrite growth and branching.^53^ Since ROCK inhibition can lead to more stable growth cones, it is unclear how VinTS FRET changes in response to deactivation of myosin-II in neurons. Future work may be focused on identifying when neurons form adhesions during maturation in culture, how VinTS FRET and tension changes at these locations, and whether longer-term myosin II inhibition promotes the formation of such stable adhesions.

Unlike the distribution of vinculin reported by others, we did not observe a distinct punctate distribution of VinTS at the growth cones. For example, in dorsal root ganglia neurons plated on laminin-coated substrates, FAK and vinculin are distributed in punctate clusters and are localized in the central body of growth cones and at the filopodial tips.^27^ These clusters have been defined as point contacts, which are small integrin clusters containing adhesion sites that differ in size and distribution than those in non-neuronal focal adhesions. Point contacts contain both vinculin and $\beta_{1}$ integrins, implying that point contacts are mature adhesion sites.^27^ In PC12 cells plated on laminin-coated substrates,^28^ vinculin is localized in filopodia, lamellipodia, and at branch points of neurites. Vinculin exhibited punctate staining in the region of growth cones closer to the neurite.^28^ Differences in vinculin distribution may be due to differences in the cell types studied, such as PC12 cells,^28^ dorsal root ganglia neurons,^27^^,^^29^ or primary cortical neurons in this study. In addition, the distribution of vinculin may depend on the maturity of the neurons and the stability of the adhesions within the growth cones.

Since we were unable to locate punctate regions with increased VinTS expression, we based our results on the average FRET efficiency of the whole growth cone region. However, FRET efficiency was not always uniform, and pixels with both high and low FRET efficiency could be found within the growth cone regions (Fig. S2 in the Supplemental Materials). The cumulative distribution function of FRET efficiency on a pixel-by-pixel basis was shifted to lower FRET in the growth cones expressing VinTS compared with those expressing VinTL [Fig. 8(c)]. By collecting time-lapse data from representative growth cones imaged between DIV 5 to 8, we also show that the growth cone morphology and mean FRET efficiency can change dynamically as a function of time (Figs. 10–12). These changes may reflect attachment and detachment of the growth cone from the substrate during migration. Thus, it is possible that vinculin distribution and tension will change during neurite migration before the formation of stable adhesion sites.

One limitation of this study lies in the averaging of a very non-homogeneous and dynamic population of pixels within the growth cones. In epithelial cells, the analysis may be confined to stable focal adhesion sites where vinculin is highly expressed and is expected to be under tension. Our inability to identify such sites in the growth cones prevented us from performing a one-on-one comparison between VinTS and VinTL at specific locations where vinculin is expected to be under tension. Instead, our analysis included regions where VinTS may not have been under tension to begin with, potentially resulting in the very small difference between the FRET efficiency of VinTS and VinTL. Our inability to identify stable adhesion sites also confounds the effect of the ROCK inhibitor Y-27632 that we measured by considering all pixels within the treated growth cones and by comparing different populations of treated and untreated neurons. In particular, sensitivity to a decrease in tension after Y-27632 treatment may be limited on a background of low tension and high FRET efficiency. Rather than measuring the effect of Y-27632 in all pixels in different populations of growth cones, the expected decrease in tension in response to myosin inhibition may have been observed by measuring VinTS FRET at the same location over time and starting at adhesion points initially under tension as may be done in epithelial cells. However, in the growth cones that we imaged at DIV 5 to 8, we were unable to identify such clear adhesions. While we could identify regions of high vinculin expression, these regions were not specifically associated with low FRET (high tension). In fact, based on our dynamic data (Fig. 10), such putative adhesion locations, or regions at which vinculin is highly expressed, seem to change within an extending growth cone such that there did not seem to be stable positions with characteristic morphology as is the case with focal adhesions in epithelial cells. Future work may, therefore, be focused on elucidating these dynamic changes and specifically tracking the movement and FRET efficiency of pixels with high vinculin expression.

In conclusion, our results provide a basis for studying the dynamics of vinculin tension in neuronal growth cones. Future studies may be aimed at further characterizing the effect of morphological dynamics on localized changes in VinTS spatial distribution and FRET efficiency. The system may be mechanically perturbed by plating the neurons on surfaces with different stiffnesses, ultimately leading to further understanding of vinculin tension dynamics in growth cones. The use of optical tweezers in conjunction with FRET force sensors has also been proposed.^54^

## Code, Data, and Materials Availability

The code used in this publication can be freely accessed and is executable through Code Ocean at https://doi.org/10.24433/CO.0316126.v1. The raw data supporting the conclusions of this article are available by request to the authors, without undue reservation.
