# Supplementary material for: Förster resonance energy transfer efficiency of the vinculin tension sensor in cultured primary cortical neuronal growth cones
Source: Neurophotonics. 2022 May 30;9(2):025002. doi: 10.1117/1.NPh.9.2.025002 (PMC9150715; doi:10.1117/1.NPh.9.2.025002)
Supplement: Supplementary file 1 [file NPh_009_025002_SD001.docx]

Supplementary Data for:

**FRET efficiency of the Vinculin Tension Sensor in Cultured Primary Cortical Neuronal Growth Cones**

**Marina A. Ayad, Timothy Mahon, Mihir Patel, Marina M. Cararo-Lopes, Ilker Hacihaliloglu, Bonnie L. Firestein, and Nada N. Boustany**

Violin Plot of Data in Fig. 8b:

To help judge the observed differences, a violin plot of the data of Fig. 8b is shown below.

Fig. S1: Violin plot of the data presented in Fig. 8b. The white dot corresponds to the median and the thick vertical line in the middle of each distribution corresponds to the interquartile range.


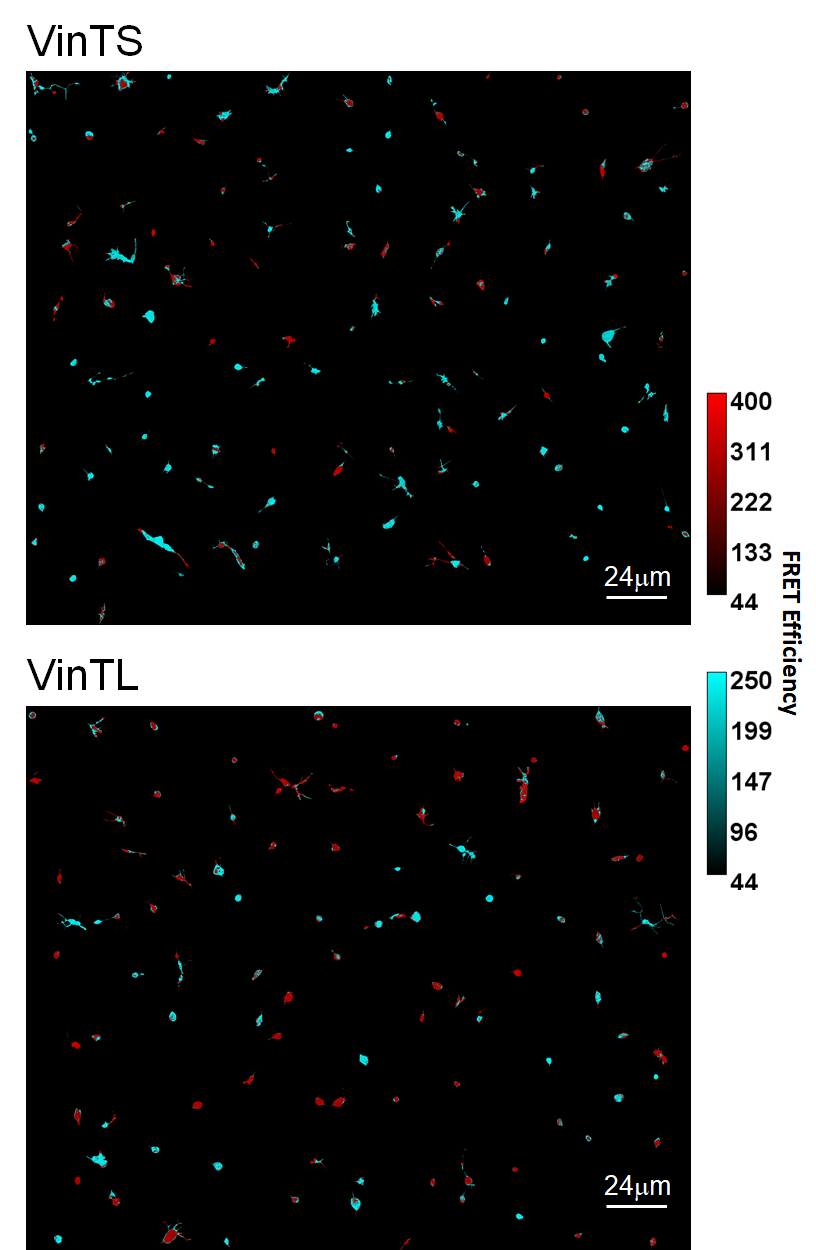
Images with segregated high and low FRET pixels:

*Figure S2: Montage showing pixels with FRET efficiency* $\geq0.25 \left( red \right)$ *and FRET efficiency <0.25 (blue) in growth cones expressing VinTS (top) or VinTL (bottom) Ratio of high(red) to low(blue) pixels is 0.66 for VinTS; 1.55 for VinTL.*

Results of Treatment with Y-27632:

For treatment with the Rho kinase inhibitor Y-27632, the aqueous stock solution (5mM) of Y-27632 (Sigma-Aldrich #68001) was dissolved in full Neurobasal medium to a final concentration of 10μM. Neurons expressing VinTS or VinTL were incubated at 37^o^C and 5% CO2 for two hours in full Neurobasal medium containing either 10μM Y-27632 or sterile water (vehicle control). Following incubation, the medium was switched to HEPES-buffered balanced salt solution (HBBSS, describe in Ref. 38 of manuscript) for cell imaging. The FRET efficiency in the growth cones was measured as described in the manuscript. Fig. S3 shows the mean FRET efficiency of the growth cones treated with Y-27632 or water and the corresponding pixel cumulative distribution functions.


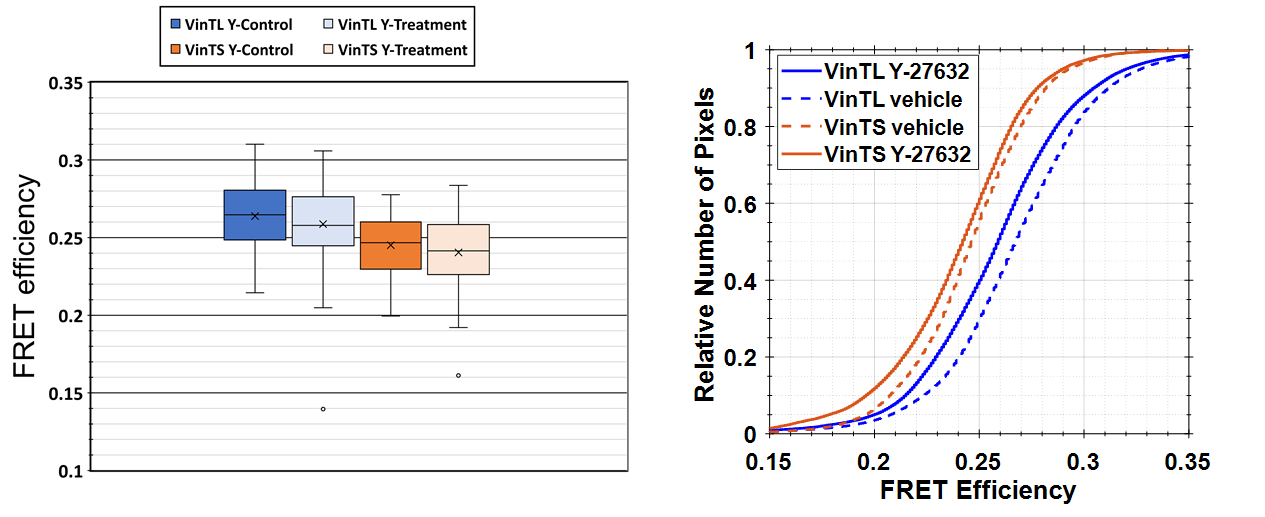
*Figure S3: Box plots (left panel) and cumulative distribution functions (right panel) of pixels in growth cones expressing VinTS (orange) or VinTL (blue) and treated with Y-27632 or vehicle (water). Mean +/- standard deviation of FRET efficiency were: 0.240 +/- 0.0256 (VinTS-Y27632), 0.245 +/- 0.0209 (VinTS-control), 0.259 +/- 0.0269 (VinTL-Y-27632) and 0.264 +/- 0.0224 (VinTL-control). The difference between the VinTS and VinTL groups was statistically significant (p< 10^-3^). However, the interaction between plasmid and treatment was not statistically significant and there was no significant difference between the treated and control groups for each plasmid (Two-way ANOVA followed by Tukey’s multiple comparisons tests).*

FRET efficiency as a function of acceptor signal:

FRET efficiency was not correlated with acceptor signal in the growth cones studied (Fig. S4).

Figure S4: Mean FRET efficiency plotted against the mean background-subtracted acceptor signal (in CMOS counts). Each point represents an individual growth cone expressing VinTS (orange) or VinTL (blue). A linear fit to the data shows no correlation between FRET efficiency and acceptor signal (R^2^= 0.01).
